# Insulin-Producing Cells Regulate the Sexual Receptivity through the Painless TRP Channel in *Drosophila* Virgin Females

**DOI:** 10.1371/journal.pone.0088175

**Published:** 2014-02-04

**Authors:** Takaomi Sakai, Kazuki Watanabe, Hirono Ohashi, Shoma Sato, Show Inami, Naoto Shimada, Toshihiro Kitamoto

**Affiliations:** 1 Department of Biological Sciences, Tokyo Metropolitan University, Tokyo, Japan; 2 Department of Anesthesia, University of Iowa, Iowa City, Iowa, United States of America; 3 Interdisciplinary Graduate Programs in Genetics and Neuroscience, University of Iowa, Iowa City, Iowa, United States of America; University of Arkansas, United States of America

## Abstract

In a variety of animal species, females hold a leading position in evaluating potential mating partners. The decision of virgin females to accept or reject a courting male is one of the most critical steps for mating success. In the fruitfly *Drosophila melanogaster*, however, the molecular and neuronal mechanisms underlying female receptivity are still poorly understood, particularly for virgin females. The *Drosophila painless* (*pain*) gene encodes a transient receptor potential (TRP) ion channel. We previously demonstrated that mutations in *pain* significantly enhance the sexual receptivity of virgin females and that *pain* expression in *pain^GAL4^*-positive neurons is necessary and sufficient for *pain*-mediated regulation of the virgin receptivity. Among the *pain^GAL4^*-positive neurons in the adult female brain, here we have found that insulin-producing cells (IPCs), a neuronal subset in the pars intercerebralis, are essential in virgin females for the regulation of sexual receptivity through Pain TRP channels. IPC-specific knockdown of *pain* expression or IPC ablation strongly enhanced female sexual receptivity as was observed in *pain* mutant females. When *pain* expression or neuronal activity was conditionally suppressed in adult IPCs, female sexual receptivity was similarly enhanced. Furthermore, both *pain* mutations and the conditional knockdown of *pain* expression in IPCs depressed female rejection behaviors toward courting males. Taken together, our results indicate that the Pain TRP channel in IPCs plays an important role in controlling the sexual receptivity of *Drosophila* virgin females by positively regulating female rejection behaviors during courtship.

## Introduction

To understand how sexual behavior is controlled by the nervous system, it is essential to identify the relevant neural circuits in the brain and elucidate how they integrate multiple sensory cues to regulate highly coordinated motor outputs. In *Drosophila melanogaster*, the sites in the central nervous system (CNS) relevant to male sexual behaviors have been extensively studied using various genetic and molecular tools [1]–[5]. In contrast, little is known about the neuronal mechanisms in the CNS underlying female mating behavior, despite the fact that females are largely responsible for the selection of a mating partner in *Drosophila* and that a mating decision by virgin females is one of the most important factors for mating success [6]–[9].

The *Drosophila painless* (*pain*) gene encodes a transient receptor potential (TRP) ion channel of the TRPA subfamily [10]. *pain* was originally identified as a gene important for thermal and mechanical nociception [11]–[13]. Further studies have revealed that *pain* is involved in a variety of neural processes including behavioral responses to wasabi [14], larval social behavior [15], negative geotaxis [16], responses to mechanical stress [17], inhibition of homosexual courtship [18], and long-term memory induced by courtship conditioning [19]. In addition, we have shown that *pain* plays a critical role in regulating sexual receptivity in *Drosophila* virgin females [20]. Specifically, *pain* mutant females have higher mating success rates than wild-type females and copulate with males earlier after males initiate courtship behavior. Considering that males court wild-type and *pain* females to the same extent, the enhanced mating success of *pain* females is most likely to be caused by increased female sexual receptivity. A GAL4-insertion in the putative 5′-flanking region of the *pain* gene, *pain^GAL4^*, drives GFP reporter expression in the larval peripheral nervous system in a pattern of the endogenous *pain* mRNA [11]. *pain^GAL4^* also drives GFP reporter expression in the adult brain and sensory neurons [12], [14], [18], [19], [20]. The enhanced female receptivity in *pain* mutants is rescued and phenocopied, respectively, by expressing the wild-type *pain* gene and *pain* RNAi using *pain^GAL4^*. Thus, the expression of *pain* in *pain^GAL4^*-positive neurons is necessary and sufficient for the Pain-mediated regulation of female sexual receptivity [20].

*pain^GAL4^* drives GFP reporter gene expression in various brain regions including the mushroom bodies (MBs), a part of the central complex (CX), and the pars intercerebralis (PI). In this study, we examined whether targeted expression of the *pain* RNAi to these *pain^GAL4^*-positive brain regions could mimic the phenotype of *pain* mutant females and enhance their sexual receptivity. Our results demonstrate that insulin-producing cells (IPCs) in the PI are critical for the Pain-mediated regulation of female sexual receptivity and that neurosecretion from IPCs negatively controls the sexual receptivity of virgin females by positively regulating their rejection responses toward courting males.

## Results

### *pain^2^* Mutant Females Copulate Earlier than Wild-type Females

**Figure 1:**
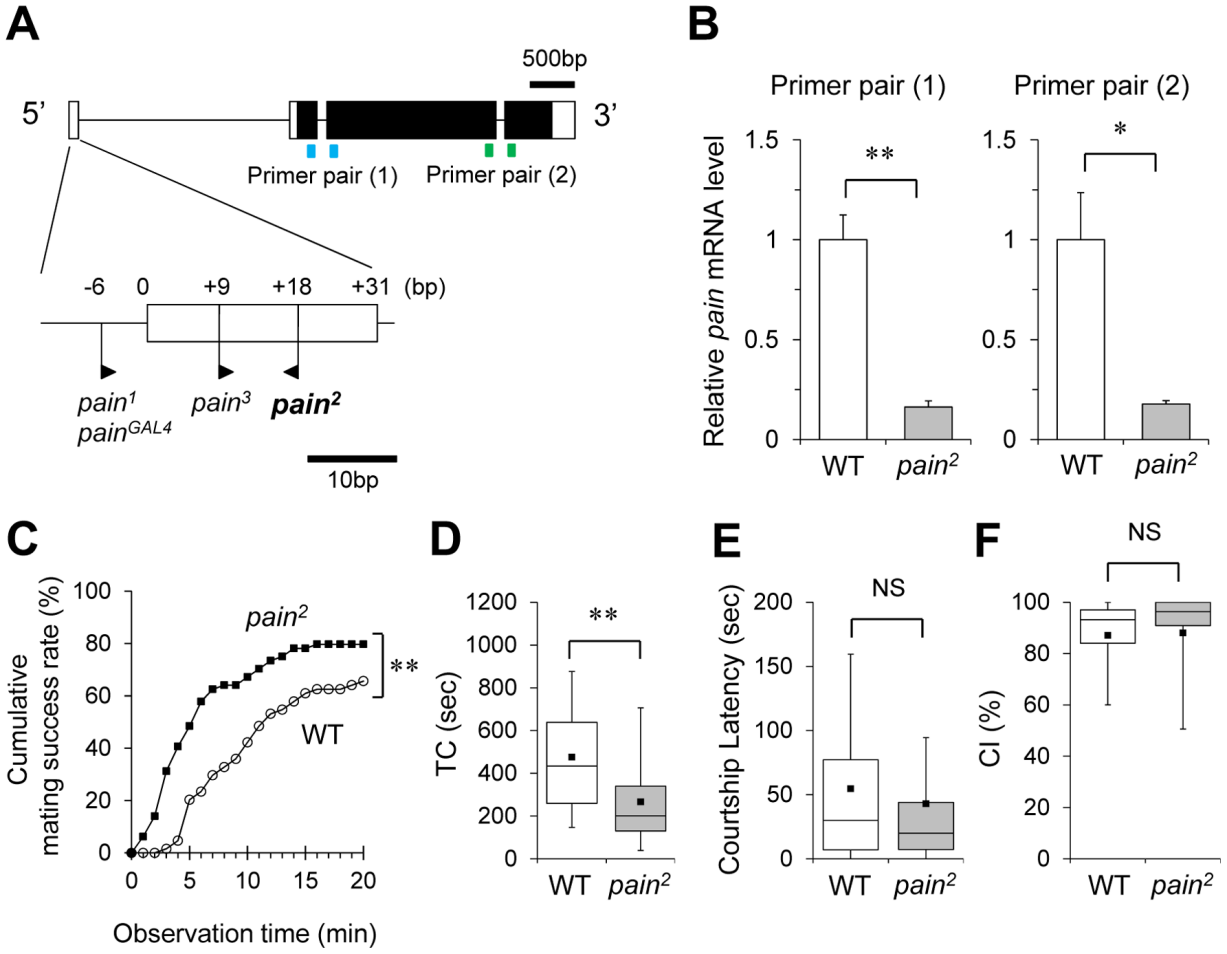
*pain^2^* females show enhanced sexual receptivity. (A) Genomic structure of the *pain* gene. White boxes represent the noncoding region. Black boxes represent the coding region. P-element insertion sites of each *pain* mutant are shown by flags. The orientation of each flag corresponds to the direction of the GAL4 sequence (*pain^GAL4^*) and GAL4-binding sequence (*pain^1^*, *pain^2^*, and *pain^3^*). Information on the genomic structure of the *pain* locus and the positions of transposon insertions was obtained from FlyBase (http://flybase.org/reports/FBgn0060296.html). Blue shows primer pair (1) and green shows primer pair (2) (see Table S1). (B–F) *pain^2^* and wild-type (Canton-S strain) females were used. WT, wild-type; *, *P*<0.05; **, *P*<0.01; NS, not significant. (B) Real-time qRT-PCR analysis of *pain* mRNA expression level. Student’s *t*-test was used for statistics. (C–F) Wild-type Canton-S males were used. (C) Cumulative mating success rate (%) in *pain^2^* (black squares) and wild-type (WT, open circles) females. The observation period was 20 min. 64 pairs were observed for each genotype. A log-rank test was used for comparison of cumulative mating success rate. **, *P*<0.01. (D) Time to copulation (TC). *N* = 42 in WT, *N* = 51 in *pain^2^*. A Mann-Whitney *U* test was used for statistical analysis. (E) Male courtship latency (sec). *N* = 64 in each genotype. A Mann-Whitney *U* test was used for statistical analysis. (F) Male courtship index (CI). *N* = 42 in WT, *N* = 51 in *pain^2^*. A Mann-Whitney *U* test was used for statistical analysis. (D–F) In each box plot, the box encompasses the interquartile range, a line is drawn at the median, and error bars correspond to the 10^th^ and 90^th^ percentiles. Each black square is the mean.

We previously reported that three *pain* mutants [*pain^1^, pain^3^,* and *pain^GAL4^* (Figure 1A)] show enhanced female sexual receptivity. Here, we have confirmed our previous finding using *pain^2^* mutant females. *pain^2^* carries an EP transposable element in the first non-coding exon (Figure 1A). The *pain^2^* mutation in females leads to a significant reduction in *pain* mRNA expression (80% reduction in females homozygous for *pain^2^* compared with wild-type females) (Figure 1B and Table S1).

The mating success rate of *pain^2^* homozygous females was significantly higher than that of wild-type females during the entire observation period (Figure 1C, log-rank test, *χ^2^* = 10.431, *P*<0.01). Next, we measured the time required for copulation after males initiate courtship behavior [time to copulation (TC)]. TC of *pain^2^* females was significantly shorter than that of wild-type females (Figure 1D). In contrast, no significant difference was detected between wild-type and *pain^2^* females in the courtship latency (the duration between the introduction of a pair of flies in the observation chamber and the first courtship) (Figure 1E), courtship index (the percentage of time spent courting during a given observation period) (Figure 1F), and general locomotion (Figure S1). These results indicate that *pain^2^* and wild-type females elicit male courtship behavior at a similar level and that the rapid copulation of virgin *pain^2^* females is due to their enhanced sexual receptivity, as was observed for *pain^1^, pain^3^,* and *pain^GAL4^* females.

### Targeted Expression of *Pain* RNAi to IPCs Enhances Female Sexual Receptivity

**Figure 2:**
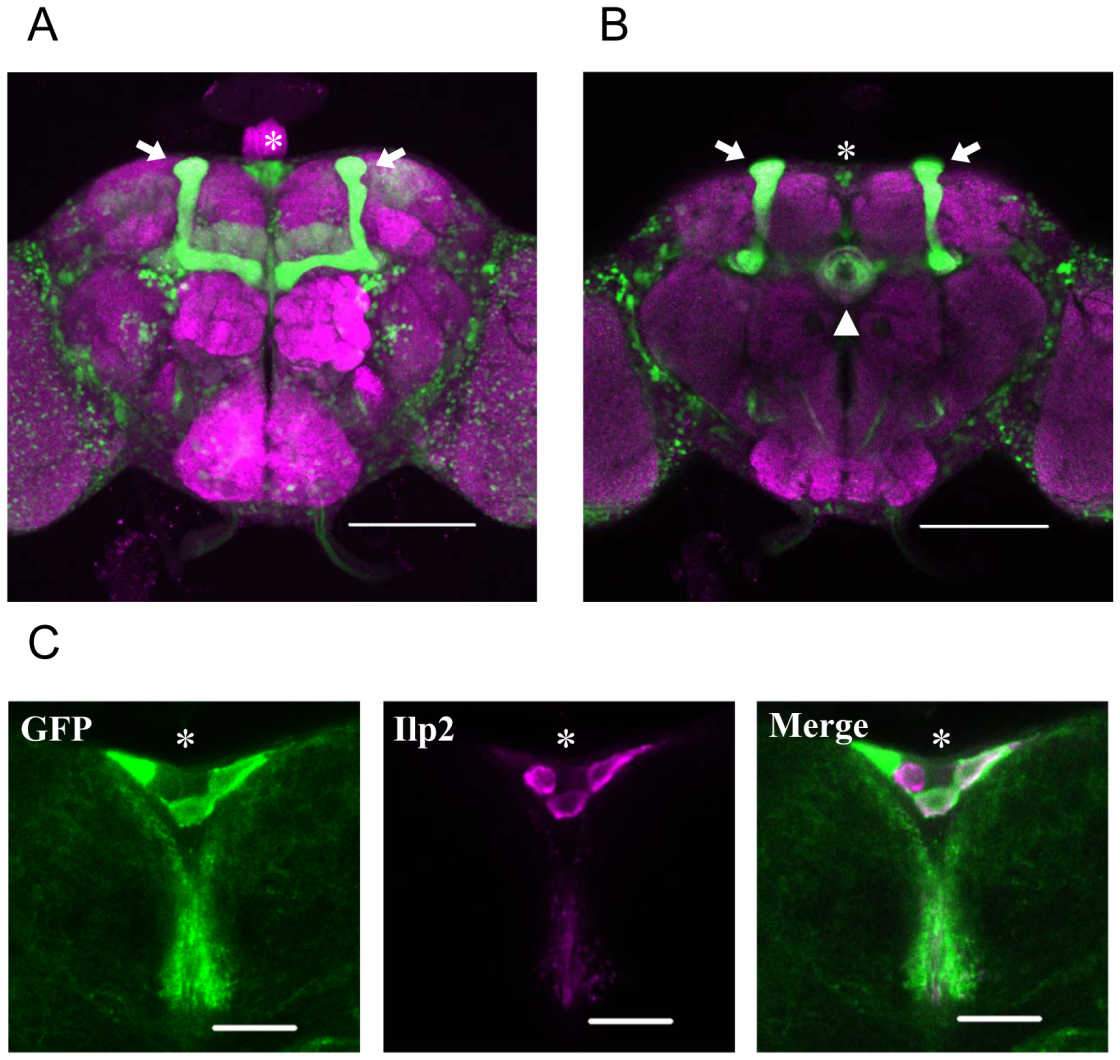
*pain^GAL4^* drives GFP reporter expression in the female brain. (A, B) Females homozygous for *pain^GAL4^* and UAS-*GFP* were used. (A) Stacked confocal image showing a frontal view of the adult brain. Scale bars represent 100 µm. Arrow, MBs; asterisk, PI. (B) Section image at the level of the EB of the adult brain. Scale bars represent 100 µm. Arrow, MBs; asterisk, PI; triangle, EB. (C) Confocal section image of *pain^GAL4^*- driven GFP in the PI neurons (green) and Ilp2 immunolabeling (magenta). *pain^GAL4^*; UAS- *mCD8::GFP* females were used. Scale bars represent 20 µm. Asterisks show the PI neurons.

We have previously reported that the expression of *pain* in *pain^GAL4^*-positive neurons is necessary and sufficient for the Pain-mediated regulation of female sexual receptivity [20]. In females, GFP reporter gene expression was driven by *pain^GAL4^* in various brain regions including the MBs, the ellipsoid body (EB) of the CX, and PI (Figure 2A and 2B) as was observed in males [19].

**Figure 3:**
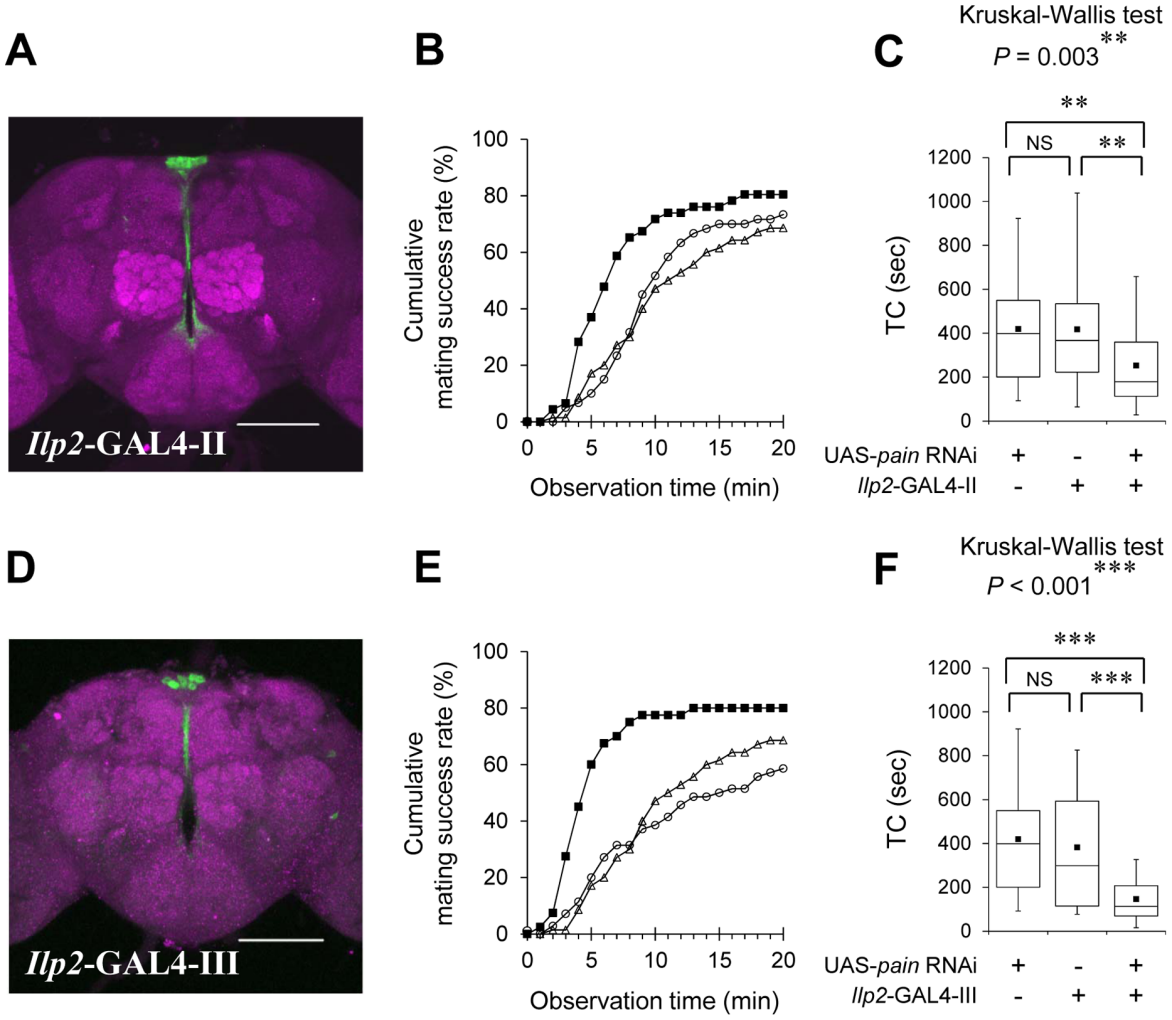
Knockdown of *pain* expression in IPCs enhances female sexual receptivity. (A, D) Stacked confocal image showing a frontal view of the adult brain in F_1_ females between UAS-*mCD8::GFP* and *Ilp2*-GAL4-II (A), or *Ilp2*-GAL4-III (D). Scale bars represent 100 µm. (B, E) Cumulative mating success rate (%) in F_1_ between GAL4 and UAS-*pain* RNAi lines (black squares), GAL4 control (open circles), and UAS control (open triangles) females. The observation period was 20 min. 40–80 pairs were observed for each genotype. (C, F) Time to copulation (TC) was measured. Non-parametric ANOVA (Kruskal-Wallis test) was used for statistical analysis. If a significant difference was evident in the Kruskal-Wallis test, a Mann-Whitney *U* test was used for pairwise comparisons. *N* = 32–48 in each genotype. **, *P*<0.01; ***, *P*<0.001; NS, not significant. (B, C) *Ilp2*-GAL4-II/UAS-*pain* RNAi, *Ilp2*-GAL4-II/+, and UAS-*pain* RNAi/+ females were used. (E, F) *Ilp2*-GAL4-III/UAS-*pain* RNAi, *Ilp2*-GAL4-III/+, and UAS-*pain* RNAi/+ females were used.

The *Drosophila insulin-like peptide 2* (*Ilp2*) gene is expressed in the IPCs, a cluster of neurons in the PI [21]–[23]. Using an anti-Ilp2 antibody as a marker of IPCs, we found that *pain^GAL4^*-positive neurons in the PI include the IPCs (Figure 2C). This raised the possibility that IPCs are involved in Pain-mediated regulation of female sexual receptivity. To examine the significance of IPCs in Pain-mediated regulation of female sexual receptivity, *pain* expression was knocked down using UAS-*pain* RNAi in combination with two independent *Ilp2*-GAL4 lines (Figure 3A and 3D). These GAL4 lines drive expression of UAS-linked genes specifically in the IPCs of the developing and adult brain [22], [23] but not in other *pain^GAL4^*-positive cells in the brain and peripheral sensory neurons (Figure S2). The effectiveness of *pain* RNAi was demonstrated by ubiquitous expression of the *pain* RNAi in females, which resulted in an approximately 70% reduction of *pain* expression relative to that of control females (Figure S3). The mating success rates of UAS-*pain* RNAi/*Ilp2*-GAL4-II and UAS-*pain* RNAi/*Ilp2*-GAL4-III females were significantly higher than those of control females (Figure 3B and 3E and Table S2). In addition, the TC of UAS-*pain* RNAi/*Ilp2*-GAL4 females was significantly shorter than those of GAL4 and UAS control females (Figure 3C and 3F). These results strongly indicate that the Pain TRP channel in IPCs serves as a negative regulator of female sexual receptivity and is necessary for its proper regulation.

**Figure 4:**
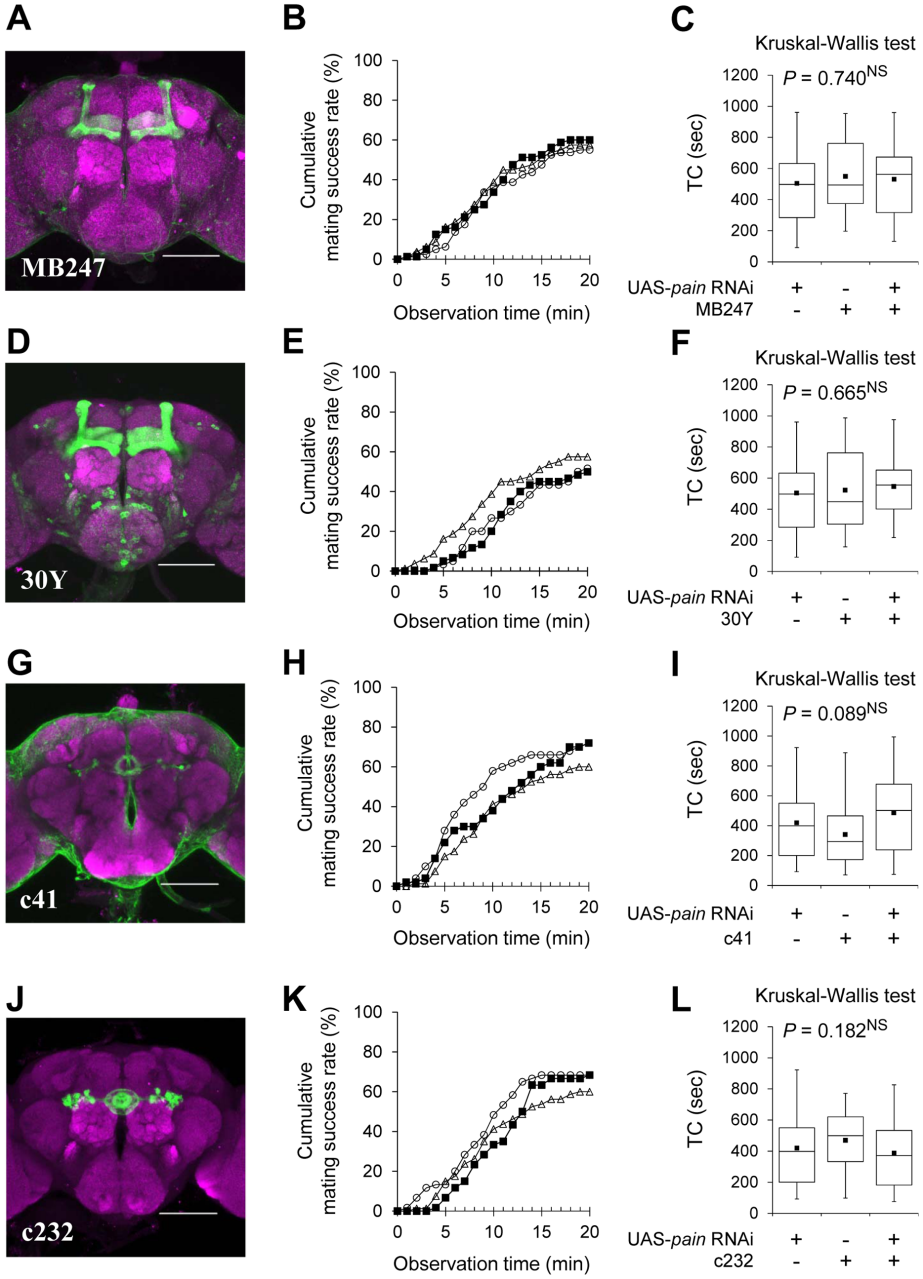
Induction of *pain* RNAi in the MBs or EB does not affect female sexual receptivity. (A, D, J) Stacked confocal image showing a frontal view of the adult brain in F_1_ females between UAS-*mCD8::GFP* and MB247 (A), 30Y (D), or c232 (J). (G) Partial stacked confocal image at the level of the EB showing a frontal view of the adult brain in F_1_ females between UAS-*mCD8::GFP* and c41. (A, D, G, J) Scale bars represent 100 µm. (B, E, H, K) Cumulative mating success rate (%) in F_1_ between GAL4 and UAS-*pain* RNAi lines (black squares), GAL4 control (open circles), and UAS control (open triangles) females. The observation period was 20 min. 50–80 pairs were observed for each genotype. (C, F, I, L) Time to copulation (TC) was measured. Non-parametric ANOVA (Kruskal-Wallis test) was used for comparisons among the three genotypes. *N* = 30–48 in each genotype. NS, not significant. (B, C) MB247/UAS-*pain* RNAi, MB247/+, and UAS-*pain* RNAi/+ females were used. (E, F) 30Y/UAS-*pain* RNAi, 30Y/+, and UAS-*pain* RNAi/+ females were used. (H, I) c41/UAS-*pain* RNAi, c41/+, and UAS-*pain* RNAi/+ females were used. (K, L) c232/UAS-*pain* RNAi, c232/+, and UAS-*pain* RNAi/+ females were used.

In contrast to two IPC-specific GAL4 drivers, combination of a UAS-*pain* RNAi line with GAL4 lines that drive expression in the MBs [MB247 and 30Y (Figure 4A and 4D)] or the EB [c41 and c232 (Figure 4G and 4J)] affected neither the mating success rate (Figure 4B, 4E, 4H, 4K, and Table S2) nor TC (Figure 4C, 4F, 4I, and 4L). Taken together, our results indicate that knockdown of *pain* expression in the IPCs specifically induces the hyper-sexual receptivity in virgin females.

### Targeted Expression of *pain* to IPCs Does not Restore Normal Sexual Receptivity in *pain* Mutant Females

**Figure 5:**
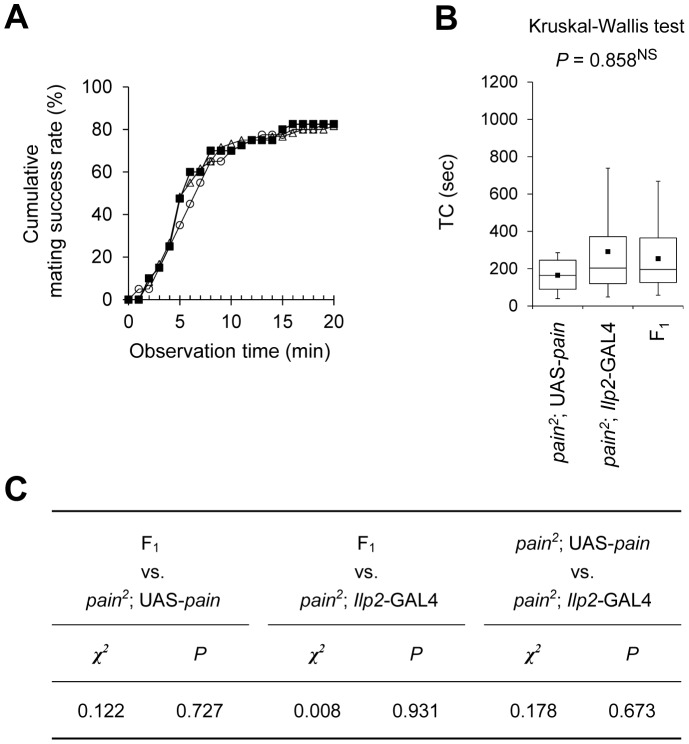
Targeted expression of wild-type *pain* to IPCs in *pain* mutant females does not restore sexual receptivity to the wild-type level. (A) Cumulative mating success rate (%) in F_1_ between *pain^2^*; UAS-*pain* and *pain^2^*; *Ilp2*-GAL4 lines (black squares), *pain^2^*; *Ilp2*-GAL4 (open circles), and *pain^2^*; UAS-*pain* (open triangles) females. The observation period was 20 min. 40–60 pairs were observed for each genotype. (B) Time to copulation (TC) was measured. Non-parametric ANOVA (Kruskal-Wallis test) was used for statistical analysis. *N* = 33–49 in each genotype. NS, not significant. (C) Statistical analysis (log-rank test) of the results shown in (A).

We previously showed that normal sexual receptivity can be restored in *pain* mutant females when expression of the wild-type *pain* gene is driven using *pain^GAL4^* with the UAS-*pain* construct [20]. We thus investigated whether expression of the wild-type *pain* gene only in IPCs is sufficient to rescue the *pain* mutant phenotype in sexual receptivity. When the wild-type *pain* was expressed in IPCs of *pain^2^* mutant females (*pain^2^*; UAS-*pain*/*Ilp2*-GAL4), their mating success rate and TC remained at the level of *pain* mutants (Figure 5). The result indicates that the targeted expression of *pain* in IPCs is insufficient to restore female sexual receptivity to the wild-type level in the *pain* mutant background.

### Female Sexual Receptivity is Enhanced by Knockdown of *pain* Expression in Adult IPCs

**Figure 6:**
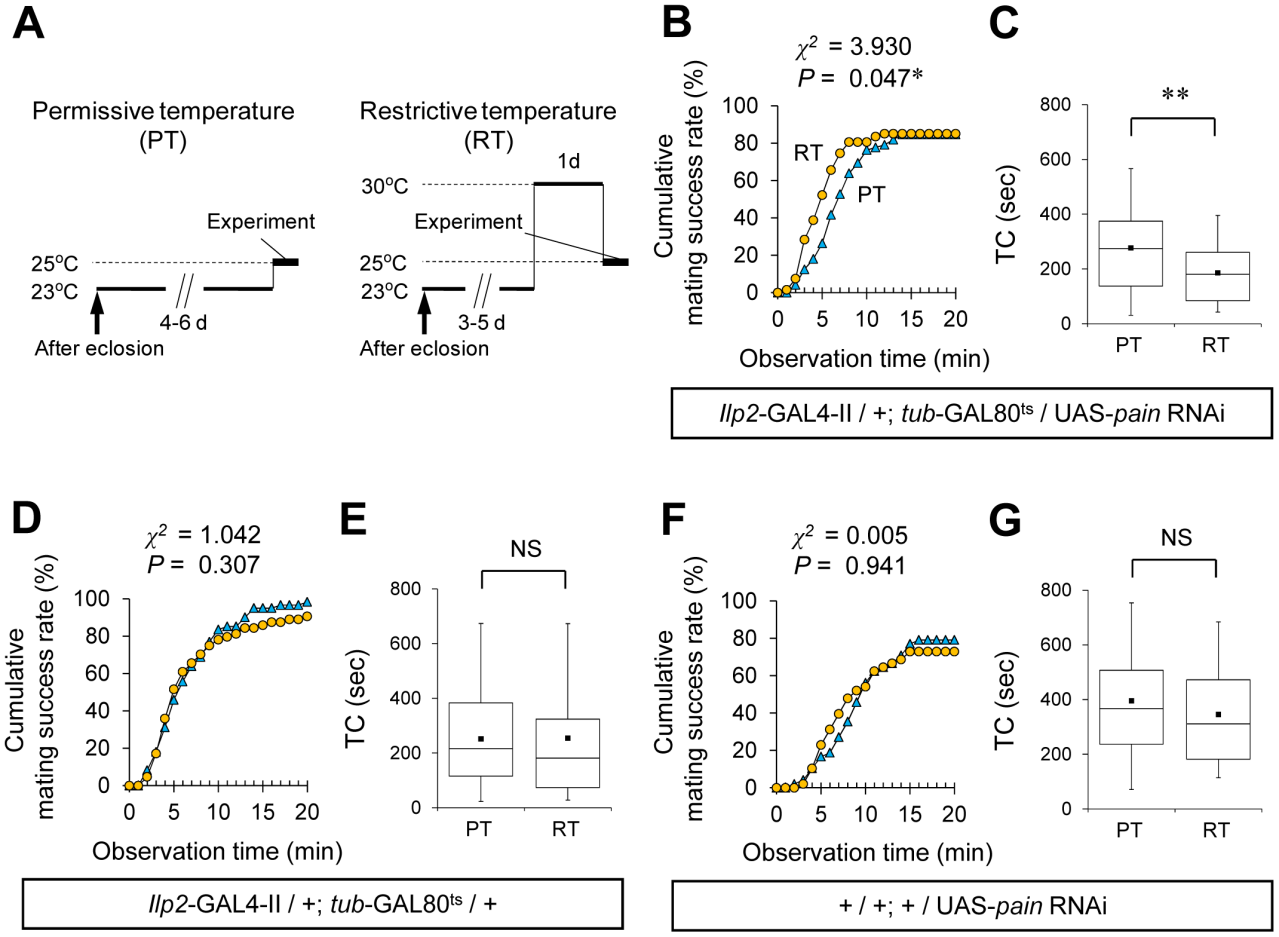
Conditional knockdown of *pain* expression in IPCs enhances female sexual receptivity. (A) Schematic diagram of temperature shift experiments. Animals were kept at 25.0±0.5°C during the embryonic, larval and pupal stages. Two temperature shift experiments (PT and RT) were performed as follows: PT, virgin females were collected and kept at the PT (23.0±0.5°C) until just before the start of experiments; RT, virgin females were collected and kept at the PT until 1 day before experiments, then they were kept at the RT (30.0±0.5°C) until just before the start of the experiments. In both PT and RT, mating behaviors were observed at 25.0±0.5°C. (B, D, F) Cumulative mating success rate (%) in *Ilp2*-GAL4-II/+; *tub*-GAL80^ts^/UAS-*pain* RNAi (B), *Ilp2*-GAL4-II/+; *tub*-GAL80^ts^/+ (D), and UAS-*pain* RNAi/+ (F) females. Virgin females of the indicated genotypes and wild-type males were used. The observation period was 20 min. 48–72 pairs were observed for each genotype. A log-rank test was used for comparison between PT (blue triangles) and RT (yellow circles). *, *P*<0.05. (C, E, G) Time to copulation (TC) in *Ilp2*-GAL4-II/+; *tub*-GAL80^ts^/UAS-*pain* RNAi (C), *Ilp2*-GAL4-II/+; *tub*-GAL80^ts^/+ (E), and UAS-*pain* RNAi/+ (G) females. *N* = 35–61 in each genotype. A Mann-Whitney *U* test was used for pairwise comparisons. **, *P*<0.01; NS, not significant.

To test whether *pain* is necessary for the acute physiological process in the regulation of female sexual receptivity, we examined the effect of selective inhibition of *pain* expression in the adult IPCs by means of the TARGET system [24]. The *tub*-GAL80^ts^ transgene used in the TARGET system encodes a ubiquitously expressed conditional GAL4 repressor that is active at the permissive temperature but not at the restrictive temperature (Figure 6A). Using *Ilp2*-GAL4/+; *tub*-GAL80^ts^/UAS-*pain* RNAi females, *pain* expression was suppressed in a temperature-dependent manner specifically in the adult IPCs. Their mating success rate at the restrictive temperature was significantly higher than that at the permissive temperature (Figure 6B) and the TC at the restrictive temperature was significantly shorter than that at the permissive temperature (Figure 6C). Control females (*Ilp2*-GAL4/+; *tub*-GAL80^ts^/+ and +/UAS-*pain* RNAi) displayed no temperature-dependent change in either the mating success rate or TC (Figure 6D–6G), showing that the temperature shift by itself does not affect female sexual receptivity. Our result indicates that *pain* expression in the adult IPCs is necessary for regulation of female sexual receptivity, and that the Pain TRP channel is acutely involved in the relevant physiological process.

### Genetic Ablation of IPCs Enhances Female Sexual Receptivity

**Figure 7:**
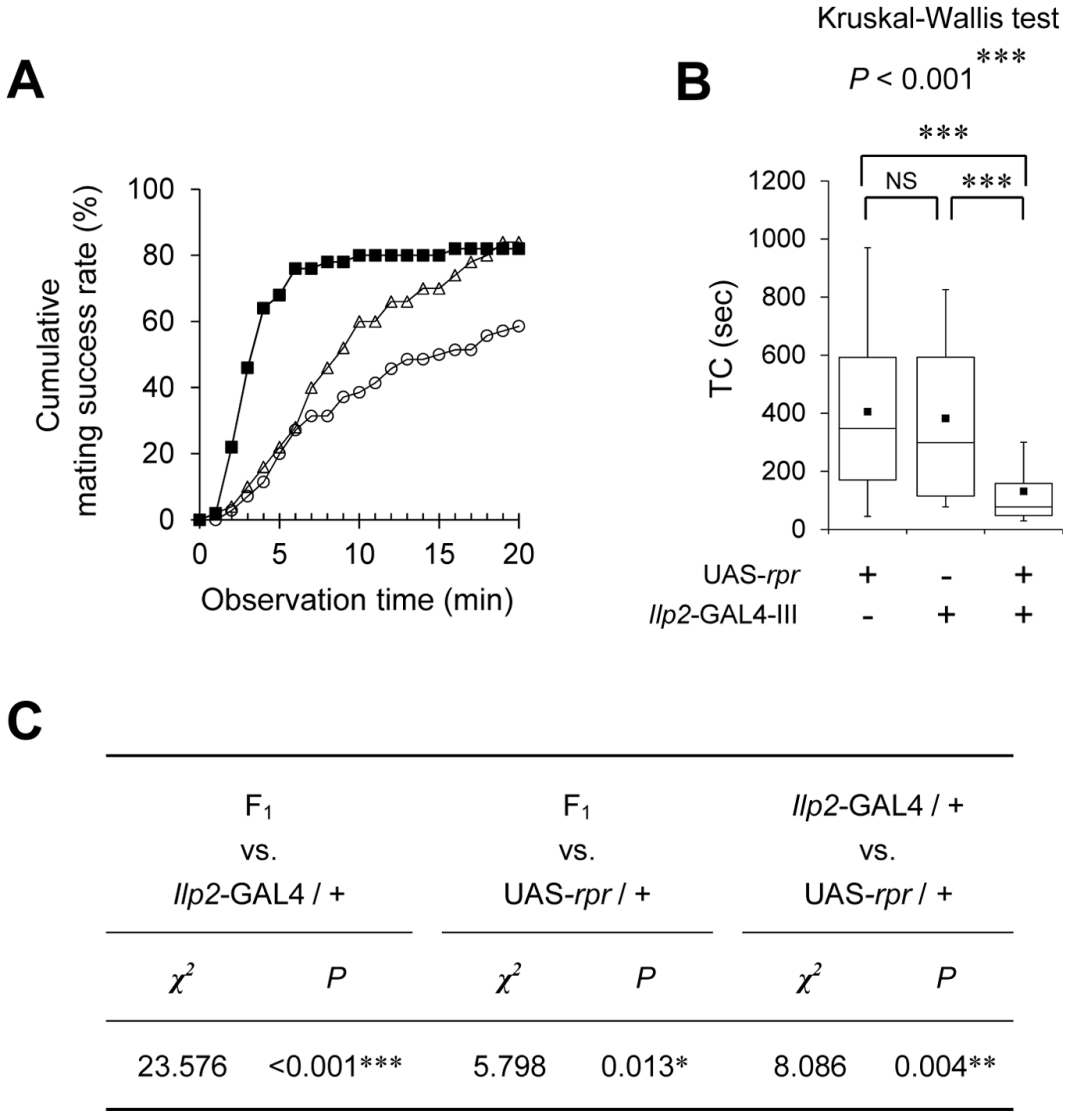
Genetic ablation of IPCs enhances female sexual receptivity. (A) Cumulative mating success rate (%) in F_1_ between UAS-*rpr* and *Ilp2*-GAL4-III lines (black squares), *Ilp2*-GAL4-III/+ (open circles), and UAS-*rpr*/+ (open triangles) females. The observation period was 20 min. 50–80 pairs were observed for each genotype. (B) Time to copulation (TC) was measured. Non-parametric ANOVA (Kruskal-Wallis test) was used for multiple comparisons and the Mann-Whitney *U* test was used for pairwise comparisons. *N* = 41–42 in each genotype. (A, B) Virgin females of the indicated genotypes and wild-type males were used. ***, *P*<0.001; NS, not significant. (C) Statistical analysis (log-rank test) of the results shown in (A).

Since the *pain*-knockdown experiments demonstrated the significance of the IPCs in the Pain-mediated regulation of sexual receptivity in virgin females, we next examined the effect of IPC ablation on mating success rate and TC. As was carried out to demonstrate the role of IPCs in lifespan regulation [23], the IPCs were ablated by expressing the pro-apoptotic gene *reaper* (*rpr*) in IPCs using *Ilp2*-GAL4. In this study, we only used F_1_ females between *Ilp2*-GAL4-III and UAS-*rpr* because F_1_ females between *Ilp2*-GAL4-II and UAS-*rpr* are lethal. The mating success rate of UAS-*rpr*/*Ilp2*-GAL4-III females was significantly higher than that of GAL4 and UAS control females (Figure 7A and 7C). Consistently, the TC for UAS-*rpr*/*Ilp2*-GAL4-III females was significantly shorter than those for GAL4 and UAS control females (Figure 7B). These results show that IPCs are required to properly suppress female sexual receptivity.

### Electrical Silencing of IPCs Enhances Female Sexual Receptivity

**Figure 8:**
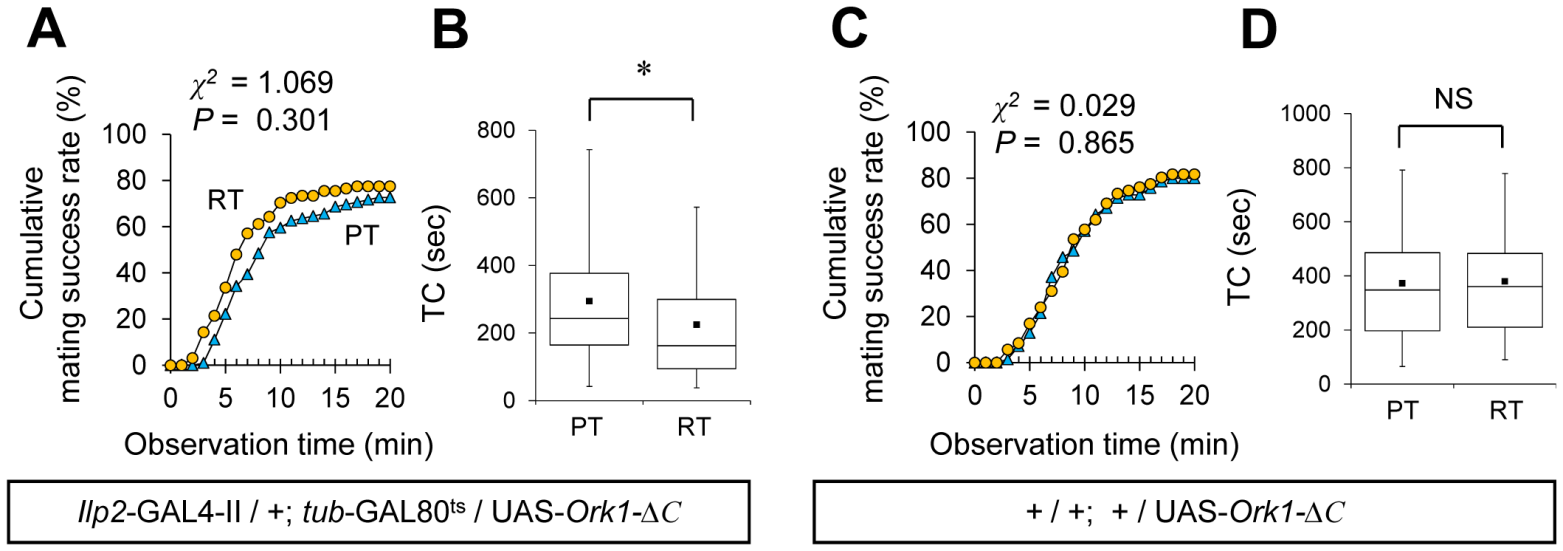
Conditional silencing of IPCs enhances female sexual receptivity. (A, C) Cumulative mating success rate (%) in *Ilp2*-GAL4-II/+; *tub*-GAL80^ts^/UAS-*Ork1-*Δ*C* (A), and +/UAS-*Ork1-*Δ*C* (C) females. Virgin females of the indicated genotypes and wild-type males were used. The observation period was 20 min. 72–132 pairs were observed for each genotype. A log-rank test was used for comparison between PT (blue triangles) and RT (yellow circles). (B, D) Time to copulation (TC) in *Ilp2*-GAL4-II/+; *tub*-GAL80^ts^/UAS-*Ork1-*Δ*C* (B), +/UAS-*Ork1-*Δ*C* (D) females. *N* = 56–104 in each genotype. A Mann-Whitney *U* test was used for pairwise comparisons. *, *P*<0.05; NS, not significant.

We next examined whether electrical silencing of IPCs affects female sexual receptivity. For this purpose, a constitutively active form of potassium channels, *Ork1-*Δ*C* [25], was used in combination with the TARGET system. In *Ilp2*-GAL4/+; *tub*-GAL80^ts^/UAS-*Ork1-*Δ*C* females, electrical activity was suppressed in the adult IPCs by shifting to the restrictive temperature during adulthood. While the mating success rate was not affected by the temperature shift (Figure 8A), the TC was significantly shortened by this treatment (Figure 8B). In control females (*Ilp2*-GAL4/+; *tub*-GAL80^ts^/+ or +/UAS-*Ork1-*Δ*C*), no temperature-dependent difference was detected in the mating success rate or TC (Figure 6D, 6E, 8C, and 8D). Thus, the shortened TC at the restrictive temperature in *Ilp2*-GAL4/+; *tub*-GAL80^ts^/UAS-*Ork1-*Δ*C* females is likely to be due to the silencing of IPCs.

### Conditional Suppression of Neurosecretion in IPCs Enhances Female Sexual Receptivity

**Figure 9:**
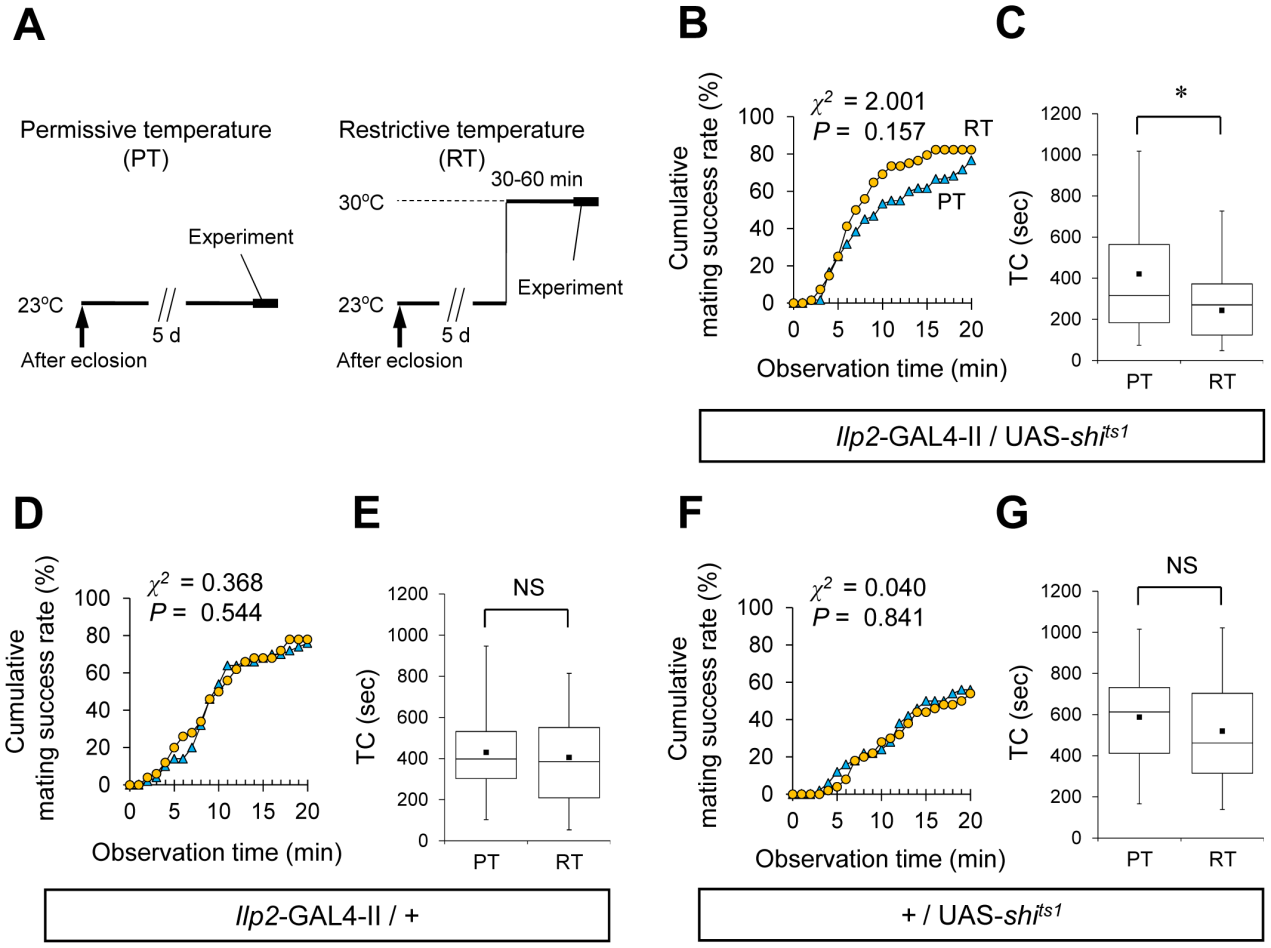
Conditional disruption of dynamin function in IPCs enhances female sexual receptivity. (A) Schematic diagrams showing the experimental paradigms of temperature shift experiments. Virgin females were collected and kept at 23.0±0.5°C (PT) until 30–60 min before the observation of mating behavior, at which point they were kept at 30.0±0.5°C (RT) until the end of the experiments. As a control, the observation of mating behavior was carried out at 23.0±0.5°C. Cumulative mating success rate (B, D, F) and TC (C, E, G) were measured for virgin females of the indicated genotypes and wild-type males. (B, D, F) A log-rank test was used for comparison between PT (blue triangles) and RT (yellow circles). *N* = 50–68. (C. E. G) A Mann-Whitney *U* test was used for pairwise comparisons. *N* = 30–56. *, *P*<0.05; NS, not significant.

Since TRP channels are calcium-permeable ion channels that can modulate neurotransmitter release and hormone secretion, the enhanced female sexual receptivity induced by *pain* inhibition could be due to reduced neurotransmission and hormone secretion from IPCs. The temperature-sensitive dynamin mutation *shibire^ts1^* (*shi^ts1^*) was used in combination with the GAL4/UAS expression system to deplete neurosecretion in a cell-type-specific and temperature-dependent manner [26]. To determine whether the suppression of neurosecretion in IPCs affects female sexual receptivity, *shi^ts1^* was expressed in IPCs using *Ilp2*-GAL4. We examined the effects of conditional disruption of dynamin function on female sexual receptivity by comparing the mating success rate and TC at the permissive and restrictive temperatures (Figure 9A). In *Ilp2*-GAL4-II/UAS-*shi^ts1^* females, no significant difference in the mating success rate was detected between the restrictive and permissive temperatures, while TC was significantly shorter at the restrictive temperature than at the permissive temperature (Figure 9B and 9C). In GAL4 (*Ilp2*-GAL4-II/+) or UAS (+/UAS-*shi^ts1^*) control females, no significant temperature-dependent difference was detected in the mating success rate or TC (Figure 9D–9G). These data suggest that neurotransmission or hormone secretion in IPCs plays a key role in maintaining female sexual receptivity at the wild-type level.

### *pain* Mutations and Knockdown of *Pain* in the IPCs Depress Female Rejection Behavior

**Figure 10:**
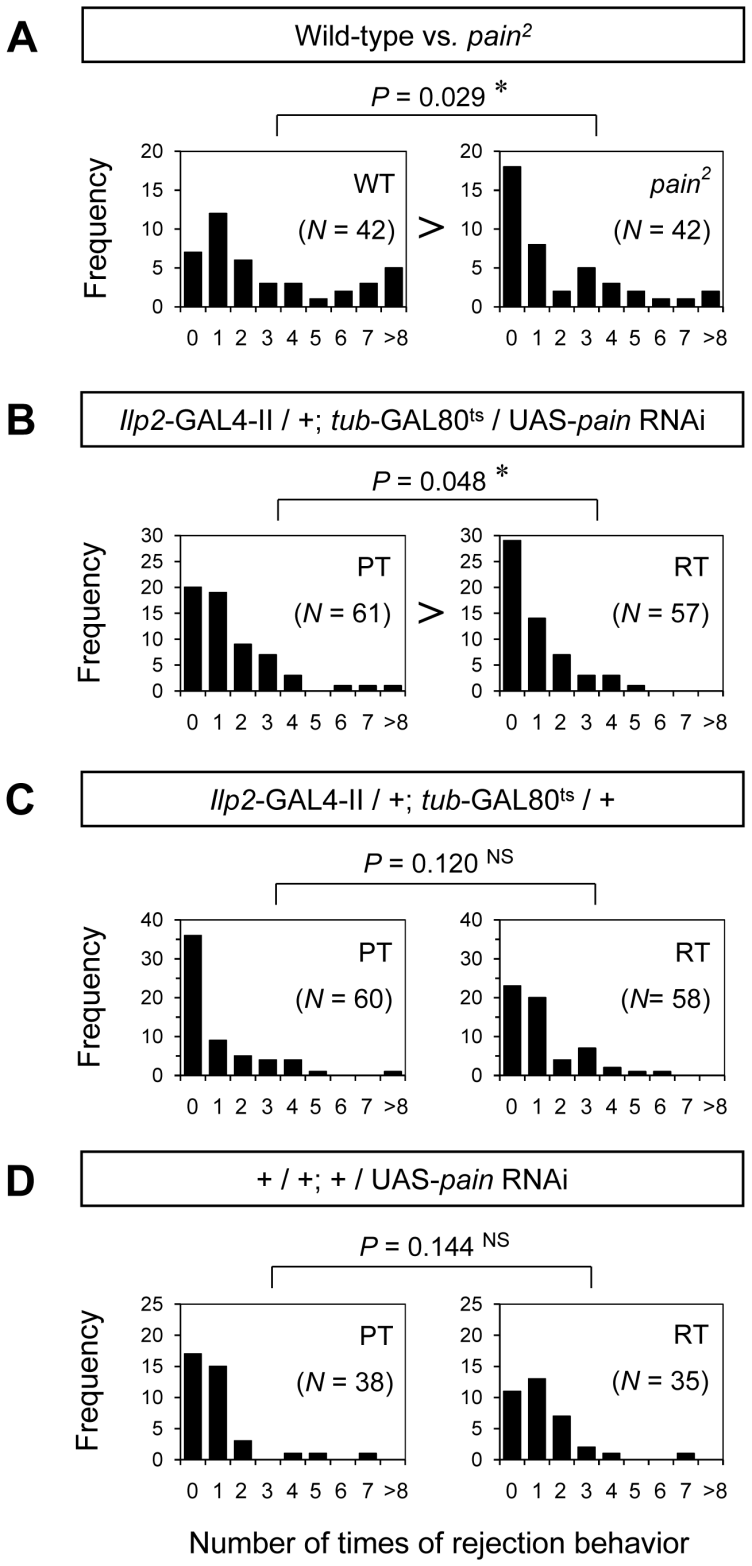
Histograms of the number of female rejections. The number of female rejection responses, defined as the number of times a female displayed rejection behavior toward a male attempting copulation, was measured. The Y-axis in each graph shows the frequency of flies in each rejection category and the X-axis categorizes female flies on the basis of the numbers of rejections in a 20 min period. Rejection frequencies of (A) wild-type (WT), *pain^2^* females, (B) *Ilp2*-GAL4/+; *tub*-GAL80^ts^/UAS-*pain* RNAi females at PT and RT, (C) *Ilp2*-GAL4/+; *tub*-GAL80^ts^/+ females at PT and RT, and (D) +/UAS-*pain* RNAi females at PT and RT are shown. A Mann-Whitney *U* test was used for pairwise comparisons. *N*, sample size; *, *P*<0.05; NS, not significant.

*pain* mutant females copulate with wild-type males earlier than wild-type females do [20]. This could be because *pain* mutations depress female rejection behavior toward courting males. To examine this possibility, we counted the number of times a female displayed one of the characteristic rejection behaviors toward a male attempting copulation (e.g., curling, decamping, or kicking) [6], [7]. The number of times *pain^2^* females rejected males was significantly lower than that of wild-type females (Figure 10A). Similar reductions in the number of rejections were observed in *pain^1^* and *pain^3^* females (Figure S4). In addition, female rejection behaviors are also depressed when *pain* RNAi is conditionally expressed in IPCs at the adult stage in *Ilp2*-GAL4/+; *tub*-GAL80^ts^/UAS-*pain* RNAi (Figure 10B). No significant differences were detected in female rejection behavior under the same conditions in control females (Figure 10C and 10D). These results suggest that *pain* activity in the IPCs is required for normal female rejection behavior. Taken together, our study indicates that TRP channels function not only as peripheral sensors for external environments but also as central sensors for internal states of animals to control complex behaviors such as female mating decision.

## Disc**u**ssion

In order to elucidate the mechanisms by which the Pain TRP channel regulates female sexual receptivity, it is essential to determine the critical sites of action for Pain. In this study, we sought to identify the *pain^GAL4^*- positive neurons that are directly involved in Pain-mediated regulation of female receptivity. For this purpose, here we used *pain^2^* mutant females instead of *pain^1^*, *pain^3^*, or *pain^GAL4^*. *pain^2^* is a unique *pain* mutant allele in that (1) unlike *pain^1^* and *pain^3^*, *pain* transcript levels are significantly reduced (Figure 1B); (2) unlike *pain^1^* and *pain^3^*, it does not induce *pain* expression when crossed to GAL4 lines because the orientation of the inserted EP element is opposite to that of *pain^1^* and *pain^3^* [11]; (3) unlike *pain^GAL4^*, it does not carry a GAL4 element. Due to these features, we were able to examine the effect of cell type-specific manipulation of *pain* expression in *pain^2^* using the GAL4/UAS system. In addition to an EP element insertion in the 5′ UTR of *pain*, *pain^2^* has a 12 kb deficiency uncovering the neighboring gene CG30427 [14]. However, it is likely that the enhanced female receptivity in *pain^2^* is solely caused by the defect in the *pain* gene because the receptivity phenotype of *pain^2^* is indistinguishable from that of other *pain* mutant alleles that are defective only in *pain*.

Among *pain^GAL4^*-positive neurons, targeted expression of *pain* RNAi in IPCs phenocopied the mutant phenotype of *pain^2^* (Figure 1) and other *pain* females [20]. This result indicates that the Pain TRP channel in the IPCs is necessary for the Pain-mediated regulation of sexual receptivity of virgin females. More specifically, our results with four distinct effector genes (UAS-*pain* RNAi, UAS-*rpr*, UAS-*Ork1-*Δ*C*, and UAS-*shi^ts1^*) in combination with *Ilp2*-GAL4 indicate that the wild-type Pain TRP channel positively controls the neuronal activity of IPCs and rejection behavior to suppress the sexual receptivity of virgin females during courtship. Because the knockdown of *pain* expression or the suppression of IPC activity during the adult stage leads to the hyper-receptive phenotype, Pain is likely to be involved in acute physiological processes in IPCs. Considering that Pain is a cation channel with extremely high Ca^2+^ permeability [13], *pain* mutations or knockdown may disturb intracellular Ca^2+^ signaling in IPCs, leading to defects in the release of neurotransmitters or hormones. The enhanced female sexual receptivity could be caused by defective neurosecretion from IPCs.

A neuropeptide, SIFamide, is expressed in four neurons in the PI of the *Drosophila* adult brain [27]. Interestingly, inhibition of SIFamide expression by RNAi in SIFamide-positive neurons, or genetic ablation of SIFamide-expressing neurons, shortens TC [27]. Because this hyper-receptive phenotype is paralleled by the knockdown of *pain* in IPCs or genetic ablation of IPCs, Pain could be involved in regulation of SIFamide secretion. However, unlike Ilp2 immunoreactivity, SIFamide immunoreactivity is not detected in *Ilp2*-GAL4-positive neurons (Figure S5). Thus, although SIFamide-expressing neurons and IPCs share common functions in controlling the sexual receptivity of females, it is unlikely that Pain regulates female sexual receptivity through SIFamide secretion.

IPCs in *Drosophila* secrete Insulin-like peptides (Ilps) and modulate various biological processes. While insulin signaling plays a central role in controlling metabolism, growth, stress resistance and lifespan [21]–[23], [28], it is also involved in regulation of behaviors (e.g., sexual dimorphism in locomotion, ethanol sensitivity, and feeding preference toward nutritive sugars) [29]–[31]. Our previous and current studies have demonstrated that targeted expression of *pain* RNAi in IPCs causes defects in long-term courtship memory in males [19] and sexual receptivity in females (this study). It is thus possible that the Pain TRP channel controls these behaviors through modulation of insulin secretion from IPCs. Although there was no obvious difference in developmental time and ovarian morphology between wild-type and *pain* mutant flies (Figure S6 and S7), all *pain* mutant flies except for *pain^1^* females displayed reduced body weight (Figure S8). This result indeed suggests the involvement of the Pain TRP channel in insulin signaling, because the phenotype is similar to that of *Ilp2*-knockout flies [28]. Interestingly, thermosensitive TRP channels are expressed in pancreatic β-cells in mammals and some of them control insulin secretion levels [32]. Thus, TRP channels could have an evolutionarily conserved role in regulation of insulin secretion.

In contrast to our finding in virgin females, Wigby et al. (2011) reported that inhibition of insulin signaling in mated females results in reduced, but not enhanced, female remating rates [33]. It is known that sperm and accessory gland proteins transferred from males to females during mating cause a variety of post-mating changes in the physiology and behavior of females. These include decreased receptivity to courting males, increased rates of ovulation and egg-laying, alterations in their longevity, and alternations in feeding and sleep patterns [34]. These changes in females could be partly attributable to mating-induced modification of the neuronal properties of IPCs and the Pain TRP channel may be involved in the mechanisms underlying the modification.

Targeted expression of wild-type *pain* in IPCs did not restore the wild-type level of female sexual receptivity in *pain* mutants (Figure 5). Thus, *pain* expression in the IPCs is necessary but not sufficient for normal Pain-mediated regulation of female sexual receptivity. It has been reported that there are three transcript variants of *pain* [35], and the longest isoform with the entire N-terminal ankyrin repeat domain is expressed in the adult brain [18]. In this study, we detected the longest isoform using a particular primer pair and confirmed that the induction of *pain* RNAi inhibits the expression of this isoform in the adult head (Figure S3). In addition, our previous study using UAS-*pain* and *pain^GAL4^* flies showed that induction of the longest isoform is sufficient to rescue the receptivity phenotype of *pain* mutant females [20]. It is thus unlikely that the failure of phenotypic rescue in *pain^2^*; UAS-*pain*/*Ilp2*-GAL4 females was due to the *pain* isoform expressed in this study. Rather, it is likely because the normal regulation of female sexual receptivity also requires the Pain TRP channel expressed in neurons other than IPCs. Our previous RNAi experiments demonstrated that the knockdown of *pain* in GABA- and acetylcholine-producing neurons leads to enhanced sexual receptivity [20]. Although the GABAergic neurons that inhibit IPCs have been identified [36], [37], there is no apparent coexpression of the markers for GABA and Ilps in the IPCs [36]. Expression of the acetylcholine-synthesizing enzyme in the PI was indicated but not confirmed [38]. These data suggest that the GABA- and acetylcholine-producing neurons distinct from IPCs are involved in the Pain-mediated regulation of female receptivity. Identification of such neurons will be important to fully understand the neuronal mechanisms underlying the Pain-mediated regulation of sexual receptivity of virgin females.

Our study has indicated that the Pain TRP channels in IPCs acutely regulate the sexual receptivity of virgin females. This raises a question concerning the physiological role of Pain in female courtship. During the early stages of courtship, the sexual receptivity of wild-type virgin females is low, and they often display rejection behaviors toward courting males (WT in Figure 10). As females repeatedly receive sensory signals of different modalities through interactions with courting males, females gradually become ready to accept a male’s copulation attempt and copulation is eventually accomplished [8], [9], [39]. There is the interesting possibility that Pain is directly involved in this modification of receptivity in response to male’s courtship behavior. The Pain TRP channels in female’s IPCs could be fully active at the initial stage of courtship and positively control female’s rejection responses toward courting males. During courtship, sensory signals produced by interactions between males and females may culminate in posttranslational modifications of the Pain TRP channels in IPCs and ultimately reduce their channel activity. Once the activity of Pain TRP channels is sufficiently reduced, as a consequence, female rejection behavior would be suppressed and females may readily accept courting males. Although the significance and molecular underpinnings of posttranslational modulation of *Drosophila* Pain remain elusive, the activities of mammalian TRP channels are known to be modulated by a wide variety of exogenous and endogenous agents and such modulations are of physiological importance [40], [41]. It would be interesting to examine whether activities of the Pain TRP channels in IPCs are modulated during courtship and how crucial such modulations are in the decision-making process for acceptance or rejection of courting males.

## Materials and Methods

### Fly Stocks

Wild-type *Drosophila melanogaster* Canton-S (CS), *pain* mutants (*pain^1^*, *pain^2^*, *pain^3^*, and *pain^GAL4^*) [11], [19], [20], UAS-*pain* RNAi [19], [20], UAS-*pain* [14], *pain^GAL4^* UAS-*GFP* [20], MB-GAL4 lines (MB247 and 30Y), EB-GAL4 lines (c232 and c41), *da*-GAL4, UAS-*rpr*, UAS-*Ork1-*Δ*C* (Bloomington stock center, 6586), UAS-*shi^ts1^* [26], UAS-*mCD8::GFP* (Bloomington stock center, 5137), *Ilp2*-GAL4-II with an *Ilp2*-GAL4 construct in the 2nd chromosome, and *Ilp2*-GAL4-III with an *Ilp2*-GAL4 construct in the 3rd chromosome, were raised on glucose-yeast-cornmeal medium at 25.0±0.5°C in a 12-h light: 12-h dark (LD) cycle. *pain* mutants, MB247, 30Y, c232, *da*-GAL4, UAS-*rpr*, UAS-*Ork1-*Δ*C*, UAS-*shi^ts1^*, *Ilp2*-GAL4-II, and *Ilp2*-GAL4-III were outcrossed for at least five generations to *white* flies with the CS genetic background. For the generation of UAS-*pain* RNAi lines, UAS-*pain* RNAi constructs were injected into *white* flies with the CS genetic background [20].

Virgin males or females were collected without anesthesia within 6 h of eclosion and maintained in vials until experiments. All the experiments except for the temperature shift experiments were carried out during daytime between Zeitgeber time (ZT) 0 and ZT5 at 25.0±0.5°C in 50–60% relative humidity.

### Observation of Mating Behavior

A pair of male and female flies was placed in an acrylic plastic observation chamber (15 mm diameter×3 mm depth) using a manual aspirator. We observed the mating behaviors for 20 min for at least 40 pairs of each genotype. We measured the mating success rate, defined as the percentage of pairs that copulated during the 20 min period after placing male and female flies together in the observation chamber. We also calculated the time to copulation (TC), courtship latency, and courtship index (CI) as described previously [20]. Wild-type CS males were used in all the observations. All flies used in the experiment were 3 to 6 days old.

### Analysis of Female Rejection Responses

Pairs of male and female flies were placed in observation chambers as described above. We observed each pair for 20 min to determine whether the female accepted or rejected the courting male that attempted copulation. In the pairs that mated within 20 min (the proportion of such pairs was more than 65% for all conditions tested), the number of times a female displayed rejection behavior toward male attempting copulation was counted. CS males were used in all the observations. All flies used in the experiment were 4 to 6 days old.

### Real-time Quantitative Reverse Transcription PCR (qRT-PCR)

Total RNA was isolated from approximately 30 female fly heads of each genotype using an RNeasy Mini Kit (QIAGEN). cDNA was synthesized by carrying out a reverse transcription reaction using a QuantiTect Reverse Transcription Kit (QIAGEN). Real-time quantitative PCR was carried out using SYBR Premix Ex Taq (Takara Bio Inc.) and a Chromo 4 Detector (MJ Research, Hercules, CA). The mean (± SEM) relative *pain* mRNA level for data resulting from four independent assays was calculated as described previously [19]. The primer sequences used for real-time qRT-PCR are shown in Table S1.

### Immunohistochemistry and Microscopy

Adult brains were stained with a mouse anti-Bruchpilot antibody (1∶20) (The Developmental Studies Hybridoma Bank at the University of Iowa, nc82), a rabbit anti-Ilp2 antibody (1∶2000) donated by T. Nishimura (RIKEN CDB, Japan) [42], and a rabbit anti-SIFamide antibody (1∶1000) donated by J. A. Veenstra (Univ. of Bordeaux, France). Alexa Fluor 568 anti-mouse IgG or anti-rabbit IgG (Invitrogen) was used as the secondary antibody (1∶1000). Fluorescence was observed using a confocal microscope (Carl Zeiss LSM710). For confocal microscopy, Z sections were collected at 1 µm intervals and processed to construct projections through an extended depth of focus.

### Statistical Analysis

In most cases, data from the courtship latency, TC, CI, general locomotion, and the number of times a female displayed rejection behavior were not distributed normally. Thus, we carried out a log transformation of courtship latency, TC, general locomotion, and the number of times a female displayed rejection behavior, and an arcsine transformation of CI. However, the transformed values did not show a normal distribution. Thus, we used the non-parametric ANOVA (Kruskal-Wallis test) for multiple comparisons and the Mann-Whitney *U* test for pairwise comparisons. The log-rank test was used for comparisons of the cumulative mating success rate. We used computer software (PASW Statistics 18) for these tests.

## Supporting Information

Figure S1 **General locomotion in wild-type (WT) and** ***pain^2^*** **females.** 3- to 5-day-old single virgin females were used for quantification of general locomotion as described previously [20]. Total distance moved (mm) was used as an index of general locomotion. Females were videotaped for 10 min. Traces were generated and total distance moved was calculated using Move-tr/2D 7.0 (Library Co., Tokyo, Japan). *N* = 40 in each genotype. NS, not significant.
(PDF)

Figure S2 **Sensory neurons visualized using GFP were observed in the legs (A, B), wings (C, D), reproductive tract (E, F), and heads (G, H) of** ***Ilp2*** **-GAL4-III/UAS-** ***mCD8::GFP*** **(A, C, E, G) and** ***pain^GAL4^*** **UAS-** ***GFP*** **(B, D, F, H) females.** Arrowheads show the second antennal segment. Arrows show the maxillary palp. Triangles show taste neurons.
(PDF)

Figure S3 **Real-time qRT-PCR analysis of** ***pain*** **mRNA expression levels.** *da*-GAL4/+, +/UAS-*pain* RNAi, and *da*-GAL4/UAS-*pain* RNAi females were used. Primer pair (1) was used. Mean ± SEM values were calculated for quadruplicated data. For multiple comparisons of relative *pain* mRNA levels among genotypes, one-way ANOVA with post-hoc Tukey’s HSD test was used. *, *P*<0.05; **, *P*<0.01; NS, not significant.
(PDF)

Figure S4 **The number of female rejection responses, defined as the number of times a female displayed rejection behavior toward a male attempting copulation, was measured.** The Y-axis in each graph shows the frequency of flies in each rejection category and the X-axis categorizes female flies on the basis of the number of rejections in a 20 min period. Rejection frequencies of wild-type (WT), *pain^1^*, and *pain^3^* females are shown. *N*, sample size; *, *P*<0.05; **, *P*<0.01.
(PDF)

Figure S5 **Immunolabeling of SIFamide and Ilp2 in the female brains.** (A) Confocal section image of SIFamide immunolabeling (magenta) and *Ilp2*-GAL4-driven GFP (green). (B) Confocal section image of Ilp2 immunolabeling (magenta) and *Ilp2*-GAL4-driven GFP (green). (A, B) F_1_ females generated between UAS-*mCD8::GFP* and *Ilp2*-GAL4-II or -III were used. Scale bars present 20 µm.
(PDF)

Figure S6 **Developmental time in wild-type and** ***pain*** **mutants.** Twenty virgin males and females (3 days old) were crossed in a food vial and their embryos were allowed to hatch. Second instar larvae were collected and transferred 60 per vial on standard food. Newly emerged flies were counted every day after the initiation of eclosion. (A) Egg-to-pupa developmental time. (B) Egg-to-adult developmental time.
(PDF)

Figure S7 **Ovary morphology in wild-type and** ***pain*** **mutants.** Newly emerged virgin females were collected within 8 h of eclosion. In each female, a pair of ovaries was dissected in PBS. It was mounted in a watch glass containing PBS. (A) The digital images of a pair of ovaries in wild-type and *pain* mutants. They were obtained by merging several differently focused images together using a software (Helicon Focus 5.3 Pro). Each section image was obtained by a digital camera (Nikon Digital Sight DS-Fi1). Scale bar, 200 µm; WT, wild-type. (B) Ovary size (µm^2^) in wild-type and *pain* mutants. Each size of a pair of ovaries dissected from a female was measured by an imaging software (Nikon NIS Elements ver. 4.0), and the average value was calculated from a pair of ovaries. Ten females were used for each genotype. We used a Mann-Whitney *U* test for pairwise comparisons (WT vs. *pain* mutants). WT, wild-type; NS, not significant.
(PDF)

Figure S8 **Body weight in wild-type and** ***pain*** **mutants.** Newly emerged males and females were briefly anaesthetized on ice and total body weight of a population of flies (10 males or females) was measured in each genotype. We replicated body weight measurements ten times and used a Mann-Whitney *U* test for pairwise comparisons (WT vs. *pain* mutants). WT, wild-type; **, *P*<0.01; ***, *P*<0.001; NS, not significant.
(PDF)

Table S1 **List of real time qRT-PCR primers.**
(PDF)

Table S2 **Statistical analysis (log-rank test) of the results shown in** **Figures 3** **and** **4** **.**
(PDF)
