# Supplementary figures and images for: Insulin-Producing Cells Regulate the Sexual Receptivity through the Painless TRP Channel in Drosophila Virgin Females
Source: PLoS One. 2014 Feb 4;9(2):e88175. doi: 10.1371/journal.pone.0088175 (PMC3913769; doi:10.1371/journal.pone.0088175)

Supplemental Figure 1

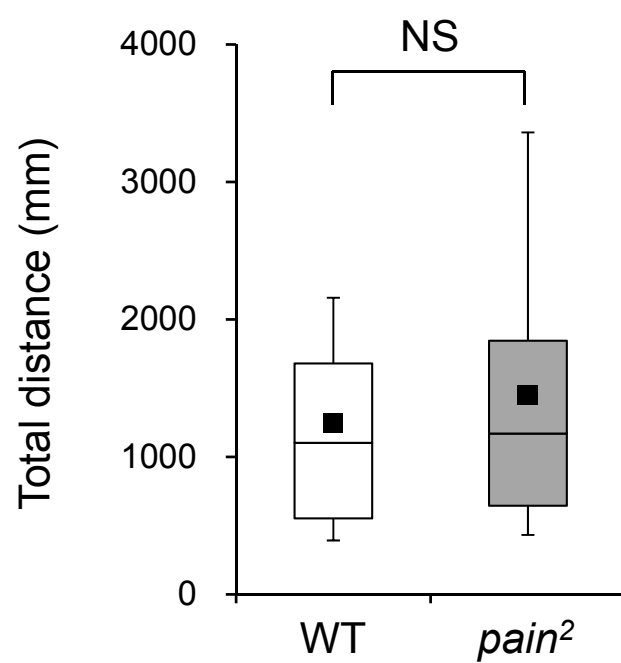

Supplement: Figure S1 — General locomotion in wild-type (WT) and pain2 females. 3- to 5-day-old single virgin females were used for quantification of general locomotion as described previously [20]. Total distance moved (mm) was used as an index of general locomotion. Females were videotaped for 10 min. Traces were generated and total distance moved was calculated using Move-tr/2D 7.0 (Library Co., Tokyo, Japan). N = 40 in each genotype. NS, not significant. (PDF) [file pone.0088175.s001.pdf]

## Supplemental Figure 2

A

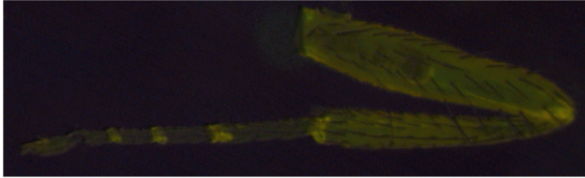

B

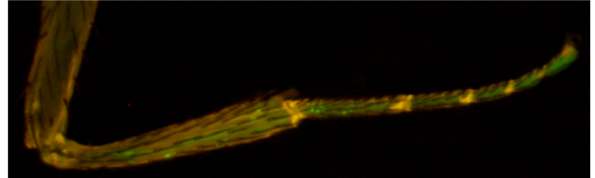

C

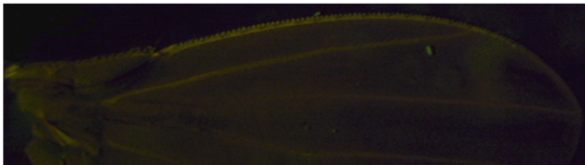

D

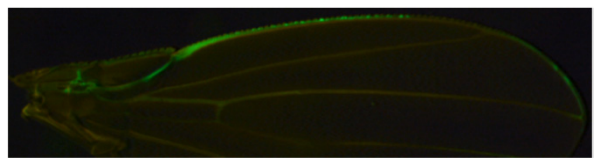

E

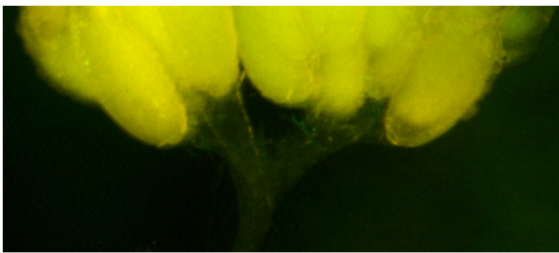

F

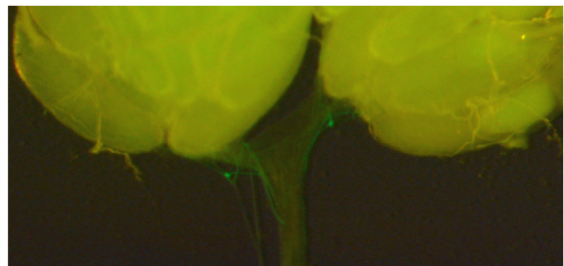

G

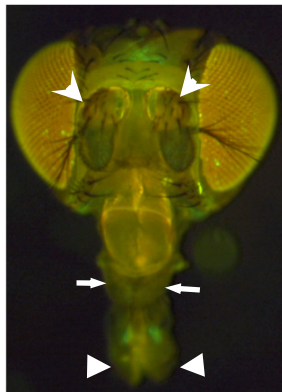

H

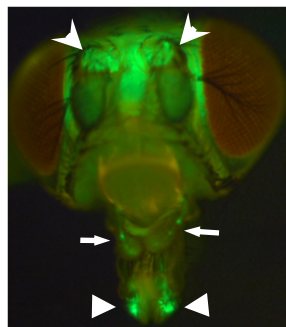

Supplement: Figure S2 — Sensory neurons visualized using GFP were observed in the legs (A, B), wings (C, D), reproductive tract (E, F), and heads (G, H) of Ilp2 -GAL4-III/UAS- mCD8::GFP (A, C, E, G) and painGAL4 UAS- GFP (B, D, F, H) females. Arrowheads show the second antennal segment. Arrows show the maxillary palp. Triangles show taste neurons. (PDF) [file pone.0088175.s002.pdf]

Supplemental Figure 3

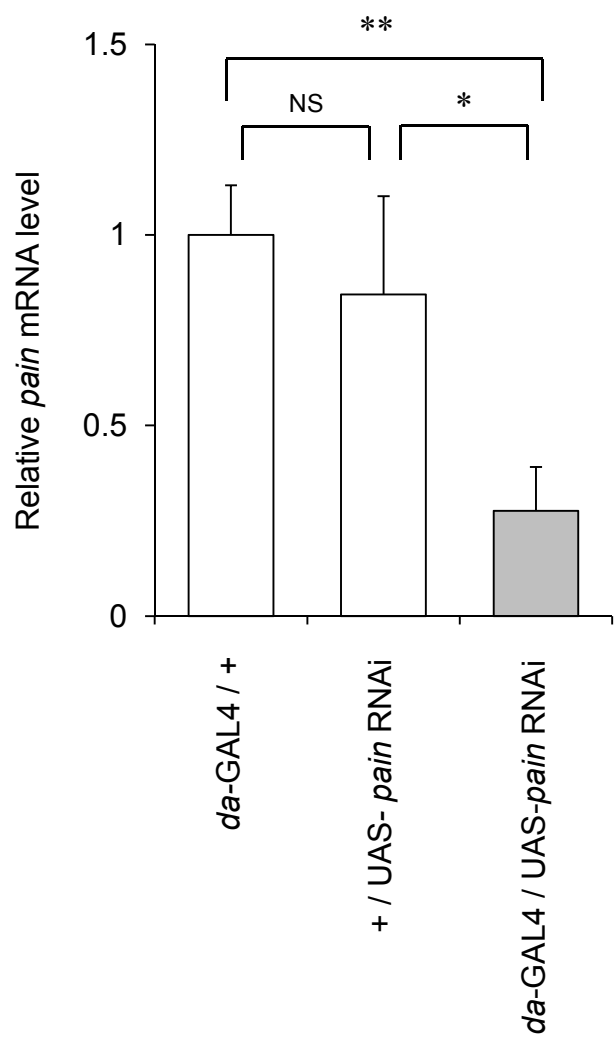

Supplement: Figure S3 — Real-time qRT-PCR analysis of pain mRNA expression levels. da-GAL4/+, +/UAS-pain RNAi, and da-GAL4/UAS-pain RNAi females were used. Primer pair (1) was used. Mean ± SEM values were calculated for quadruplicated data. For multiple comparisons of relative pain mRNA levels among genotypes, one-way ANOVA with post-hoc Tukey’s HSD test was used. *, P<0.05; **, P<0.01; NS, not significant. (PDF) [file pone.0088175.s003.pdf]

Supplemental Figure 4

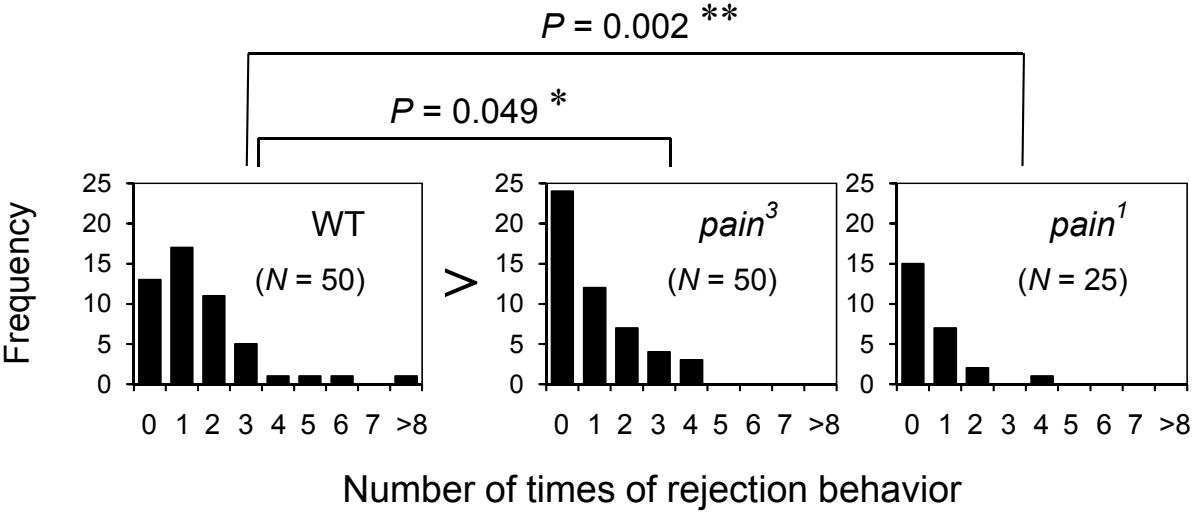

Supplement: Figure S4 — The number of female rejection responses, defined as the number of times a female displayed rejection behavior toward a male attempting copulation, was measured. The Y-axis in each graph shows the frequency of flies in each rejection category and the X-axis categorizes female flies on the basis of the number of rejections in a 20 min period. Rejection frequencies of wild-type (WT), pain1, and pain3 females are shown. N, sample size; *, P<0.05; **, P<0.01. (PDF) [file pone.0088175.s004.pdf]

Supplemental Figure 5

**A**

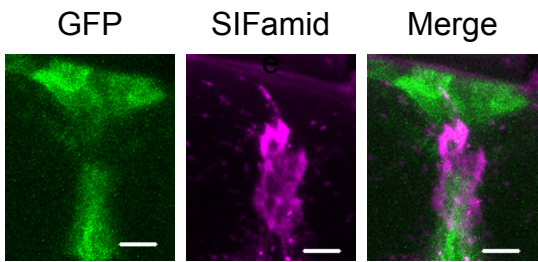

**B**

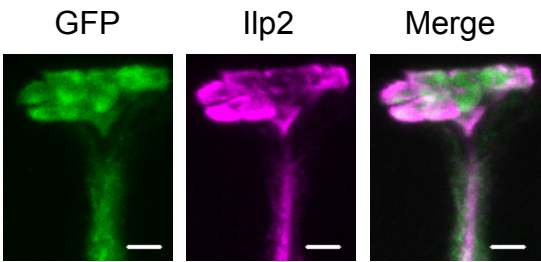

Supplement: Figure S5 — Immunolabeling of SIFamide and Ilp2 in the female brains. (A) Confocal section image of SIFamide immunolabeling (magenta) and Ilp2-GAL4-driven GFP (green). (B) Confocal section image of Ilp2 immunolabeling (magenta) and Ilp2-GAL4-driven GFP (green). (A, B) F1 females generated between UAS-mCD8::GFP and Ilp2-GAL4-II or -III were used. Scale bars present 20 µm. (PDF) [file pone.0088175.s005.pdf]

Supplemental Figure 6

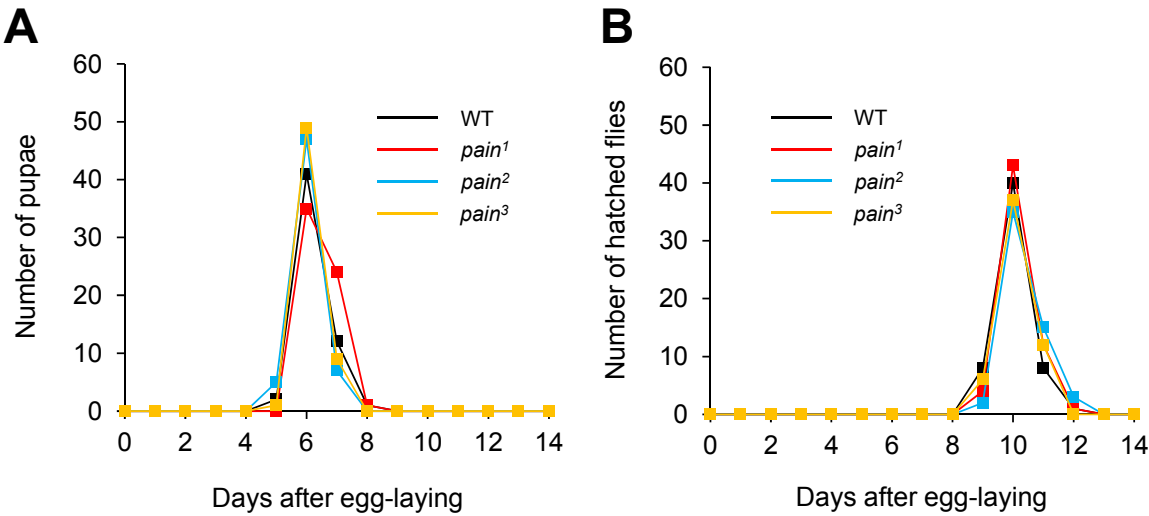

Supplement: Figure S6 — Developmental time in wild-type and pain mutants. Twenty virgin males and females (3 days old) were crossed in a food vial and their embryos were allowed to hatch. Second instar larvae were collected and transferred 60 per vial on standard food. Newly emerged flies were counted every day after the initiation of eclosion. (A) Egg-to-pupa developmental time. (B) Egg-to-adult developmental time. (PDF) [file pone.0088175.s006.pdf]

## Supplemental Figure 7

**A**

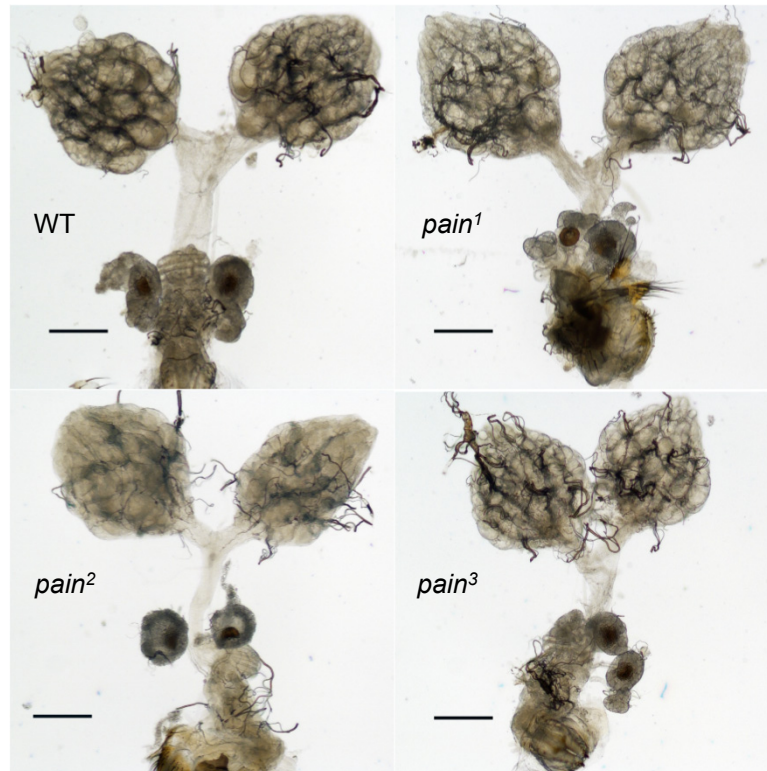

**B**

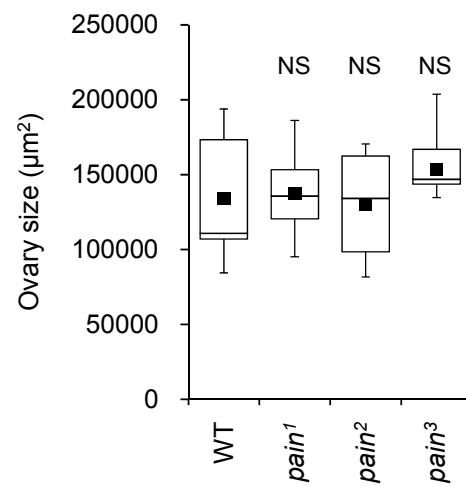

Supplement: Figure S7 — Ovary morphology in wild-type and pain mutants. Newly emerged virgin females were collected within 8 h of eclosion. In each female, a pair of ovaries was dissected in PBS. It was mounted in a watch glass containing PBS. (A) The digital images of a pair of ovaries in wild-type and pain mutants. They were obtained by merging several differently focused images together using a software (Helicon Focus 5.3 Pro). Each section image was obtained by a digital camera (Nikon Digital Sight DS-Fi1). Scale bar, 200 µm; WT, wild-type. (B) Ovary size (µm2) in wild-type and pain mutants. Each size of a pair of ovaries dissected from a female was measured by an imaging software (Nikon NIS Elements ver. 4.0), and the average value was calculated from a pair of ovaries. Ten females were used for each genotype. We used a Mann-Whitney U test for pairwise comparisons (WT vs. pain mutants). WT, wild-type; NS, not significant. (PDF) [file pone.0088175.s007.pdf]

Supplemental Figure 8

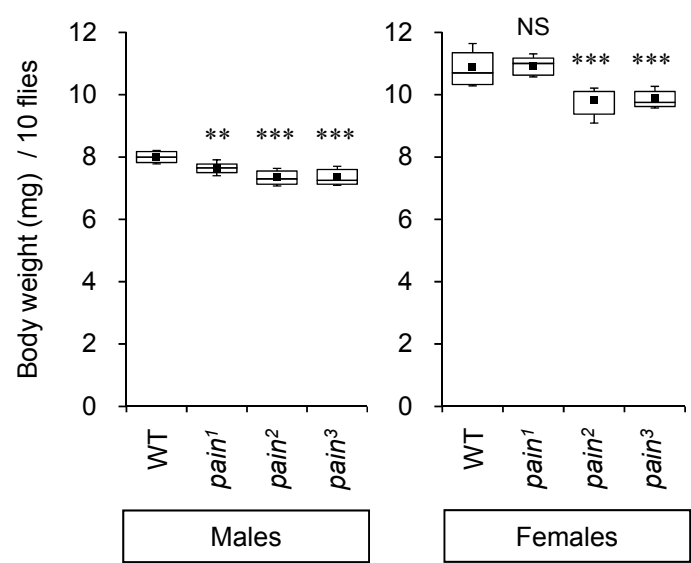

Supplement: Figure S8 — Body weight in wild-type and pain mutants. Newly emerged males and females were briefly anaesthetized on ice and total body weight of a population of flies (10 males or females) was measured in each genotype. We replicated body weight measurements ten times and used a Mann-Whitney U test for pairwise comparisons (WT vs. pain mutants). WT, wild-type; **, P<0.01; ***, P<0.001; NS, not significant. (PDF) [file pone.0088175.s008.pdf]
