# Supplementary material for: Insulin-Producing Cells Regulate the Sexual Receptivity through the Painless TRP Channel in Drosophila Virgin Females
Source: PLoS One. 2014 Feb 4;9(2):e88175. doi: 10.1371/journal.pone.0088175 (PMC3913769; doi:10.1371/journal.pone.0088175)
Supplement: Table S1 — List of real time qRT-PCR primers. (PDF) [file pone.0088175.s009.pdf]

Supplemental Table 1

Table S1. List of real time qRT-PCR primers

Gene name	Forward primer	Reverse primer
pain [primer pair (1)]	CACTCTCAACACCAGGTTGTC	AGGTTTCCTGGATCCCTAGAG
pain [primer pair (2)]	GTTCAACCTTTTGAACGGTCTTG	TAAGGACGTTGGTTCTGCAAATG
rp49	AAGATCGTGAAGAAGCGCAC	TGTGCACCAGGAACTTCTTG
