# Supplementary material for: Insulin-Producing Cells Regulate the Sexual Receptivity through the Painless TRP Channel in Drosophila Virgin Females
Source: PLoS One. 2014 Feb 4;9(2):e88175. doi: 10.1371/journal.pone.0088175 (PMC3913769; doi:10.1371/journal.pone.0088175)
Supplement: Table S2 — Statistical analysis (log-rank test) of the results shown in Figures 3 and 4 . (PDF) [file pone.0088175.s010.pdf]

# Supplemental Table 2

Table S2. Pairwise comparisons of mating success rate

| | $F_1$ vs. GAL4/+ | | $F_1$ vs. *pain* RNAi/+ | | GAL4/+ vs. *pain* RNAi/+ | |
|---|---|---|---|---|---|---|
| GAL4 lines | $\chi^2$ | *P* | $\chi^2$ | *P* | $\chi^2$ | *P* |
| (1) *Ilp2*-GAL4-II | 4.939 | 0.026* | 12.554 | <0.001*** | 2.739 | 0.098 |
| (2) *Ilp2*-GAL4-III | 14.993 | <0.001*** | 18.502 | <0.001*** | <0.001 | 0.993 |
| (3) MB247 | 0.395 | 0.530 | 0.023 | 0.880 | 0.213 | 0.644 |
| (4) 30Y | 0.013 | 0.909 | 1.664 | 0.197 | 1.291 | 0.256 |
| (5) c41 | 0.358 | 0.550 | 1.268 | 0.260 | 3.321 | 0.068 |
| (6) c232 | 0.130 | 0.719 | 0.700 | 0.403 | 0.258 | 0.612 |
